# The Pathological Anatomy of the Human Body

**Published:** 1847-04-01

**Authors:** 


THE PATHOLOGICAL ANATOMY OF THE HUMAN BODY. By *Julius Vogel*, M.D., Professor of Clinical Medicine at the University of Giessen. Translated from the German, with Additions. By *George E. Day*, M.A. & L.M., Cantab., &c. Illustrated with one hundred plain and coloured Engravings. London, H. Bailliere, 1847.

THE editor of the French translation of Dr. Vogel's Pathological Anatomy, justly observes, "in the age in which we live, value is only attached to that which is productive of positive and fruitful results. Medicine has not been able to escape this practical tendency; and hence, showing itself less curious than formerly in abstract speculations, it has converged more than ever towards the final end of all its efforts—the perfectioning of the art of curing." It may not to some persons appear very clear that a work, in which histology, microscopy, and chemistry play a conspicuous part, obeys the impulse to which we have referred; and yet no one who reads the treatise before us can doubt that it is eminently practical. It is not because many new terms, and especially since the researches of Schwann, Henle, and other observers of the same class, have been introduced, that morbid anatomy and pathology have changed their essential character; on the contrary, the objects pursued now, and the results aimed at, remain the same as in the days of Morgagni and Baillie; the means of investigation alone have been altered with the advance of science, and, with that advance, it may be safely affirmed, the modes of inquiry have become much more effectual, and the knowledge obtained, consequently, much more satisfactory. If it ever was a point of moment to form a natural classification of tumours; to distinguish definitely malignant from non-malignant growths; to detect in what consists the essential character of scirrhus, fungus, and other reputed specific formations; and, more than all this, if at any time it concerned the physician to know what is the series of structural changes inducing the morbid actions it is his office to control and remove, then is pathological anatomy, as now cultivated, a subject deserving the careful study of every enlightened practitioner.

In the introductory portion of his work, the author has with much judgment shown the limits, the objects, and the appliances of the important subject he so ably discusses. In tracing the relations of pathological anatomy with the individual branches of medical science, and after condemning the opposite but equally mischievous errors of an overweening confidence and an undistinguishing scepticism, Dr. Vogel proceeds to say—

"Above all, we must not be led away by such phrases as 'practical views' and 'medical experience'—terms of common use, and too often conveying an erroneous impression. The practical views of the physician are the result of a series of accurate observations elucidating the treatment of disease. The true physician may be distinguished from the empiric by this, that the latter is more or less unconscious of the grounds on which he acts; and if the experience of the empiric seems in some few cases to be more successful than science, it can only be referred to a fortunate chance directing to the right point, and probably not based on the conscious experience of a single case. In proportion as the science advances, and its cultivation is zealously carried on, so much the more will practical views and experience become the common property of all physicians who combine theory and practice; and that which was formerly regarded as the exclusive property of the medical pioneer will be open to all—will be almost the common stock of all who strive to obtain it." P. 4.

The vast importance of chemistry, especially in relation to those numerous diseases which are connected with the fluids of the animal body, is generally recognised in the present day; but, owing principally to the difficulty of the inquiry, a custom has arisen of regarding this subject as belonging to a separate science to which the term "*Pathological Chemistry*" has been given. That this disseveration must retard the medical knowledge of many important diseases, is sufficiently obvious; instances of serious, if not of fatal, mistakes, arising from this cause, are not indeed of rare occurrence; and we therefore extract the judicious observations of the author respecting the practice just noticed.

"In the investigation of delicate points connected with pathological histology, the microscope is indispensable, and the application of chemical re-agents must be observed under it. Chemical analysis is, indeed, of the greatest importance to pathological anatomy, being the only means by which we can on several points obtain the desired information. At present, much to the detriment of the science, chemical investigation is little pursued in conjunction with pathological anatomy; but assuredly the time will soon arrive, when chemical analysis will be deemed just as indispensable to the prosecution of pathologico-anatomical investigations as the microscope is at present, and when every follower of this science will consider chemical analysis so essentially requisite, that if his own time and opportunities prevent him from carrying it out, he will employ a chemist, under his immediate guidance and direction, to undertake it for him." P. 11.

The two great sources of accurate knowledge in pathological anatomy are, as the author affirms, *observation* and *experiment;* the former being the principal means of investigation as far as the human body is concerned; whilst the latter, for obvious reasons, is almost exclusively restricted to animals. It is hardly necessary to combat an objection, frequently urged in former years, that the results obtained from pathological changes induced in the lower animals, are not trustworthy when applied to man. Two of the most ordinary phenomena presented to the notice of the practitioner, the reparation of fractured bones and inflammation, have been elucidated almost exclusively, as regards their essential characters, by experiments and observations made on living animals; whilst, as concerns the restorative process in wounds of the intestines and divided arteries, although more has been learnt from the inspection of the human body than in the preceding instances, yet the most precise and satisfactory information has sprung from well-devised experiments practised on the brute creation. It is somewhat strange that this objection should ever have arisen, since no one could be found to deny that great light has been thrown upon many interesting branches of pathology by diseases occurring in the domesticated mammalia. For these reasons we are fully prepared to coincide with Dr. Vogel, that conclusions drawn from this source are not only admissible, but that comparative pathology and pathological anatomy afford as much assistance in the prosecution of this science in relation to man, as comparative anatomy does for the thorough comprehension of human anatomy and physiology.

But to give value to any mode of investigation, the facts must be multiplied and scrupulously weighed. Limited observations and hasty generalisations have been the special bane of medical science, not merely by leading to error, but by tending to throw discredit on the very means by which of all others the most assured results are to be attained. In short, that science, for such it is, in which so many of the great questions affecting civilised communities have found their solution, namely statistics, must be applied to that which lies at the botton of all enlightened medicine and surgery—pathological anatomy. True it is, that to apply the statistical method to morbid anatomy, is a much more difficult matter than to determine the mean duration of human life, or the average age at death; for, as the author observes, in proposing as a problem the question whether scirrhus and tuberculosis exclude each other, "although physicians are not likely to dispute whether or not a man is really dead, there are few points on which there is more difference of opinion than whether a tumour is to be regarded as of a scirrhous nature or not." There are not then, at present, the data requisite to enable some second Newton to give to pathology its laws and principles; an additinal reason this for all who are interested in the future progress of medicine to imitate our author. "In our science," he justly observes, "we must follow the examples set us by the astronomers, magnetists, and meteorologists (and he might have added the geologists), who continue for years to carry on the most careful general observations, and to make them public property, in the hope that the general laws which they fail to establish, will be developed by their successors."—*Introduction*, p. 18.

In the volume now translated, Dr. Vogel treats only on the generalities of morbid anatomy; a second volume will follow, in which the special department, or that relating to changes in the individual organs, will be comprised; and, as Dr. Day has undertaken the translation of this concluding part, the English reader will soon be in possession of one of the most recent and complete treatises that have appeared in relation to pathological anatomy. Although we feel it but just to the author to speak thus favourably of his well-known work, we cannot withhold the expression of our opinion, that it is valuable rather on account of presenting a comprehensive epitome of the existing knowledge, than for the extent of original observation in reference to microscopy as applied to morbid anatomy. And further, this treatise, in which the new system of pathology is, without reservation, adopted, appears to us to be particularly deficient in the application of the great discoveries made of late years in structural and philosophic anatomy, and which must, for the reason just stated, be regarded as a fundamental defect: the justice of these remarks will, we think, become apparent in the course of the present article.

The general scope of this first division of the subject will be understood by the following sketch of the order, in which the different morbid changes are discussed.

"We commence with abnormal collections of fluids in the body—of the gaseous (pneumatoses), of the aqueous (dropsies). The latter are divided in a manner that seems natural and practically important, although not hitherto adopted; namely, into serous, fibrinous, and false dropsies. Then comes a sketch of the morbid changes of the blood as far as they are at present understood. This is succeeded by a chapter on pathological epigeneses,* which from their nature occupy a very considerable space, and by a brief sketch of the changes which the tissues undergo in their physical properties, together with some remarks on the manner in which morbid changes in the elementary tissues are connected with each other. The next chapter treats of the independent organisms which occur in the human body, as causes or consequences of morbid changes (parasites). Then there is a chapter devoted to congenital pathological changes (malformations), and we conclude with a notice of the changes occurring in the body after death." P. 20.

The second Chapter contains some interesting remarks on the various forms of Dropsy; though the author has hazarded some opinions, the correctness of which appears to be somewhat doubtful. He distinguishes in this affection three distinct forms:—serous dropsy, in which the fluid is identical in its qualitative chemical composition with the serum of the blood; fibrinous dropsy, in which the fluid contains dissolved fibrin, and in its chemical composition resembles the plasma of the blood; false dropsy, in which the fluid differs essentially in its chemical composition from either of the preceding forms; this is, in fact, not dropsy at all, but merely an accumulation of a natural secretion, owing to an impediment to its exit, as of urine (hydrops renum), of bile, &c. These forms not only thus differ in the physical and chemical characters of their fluids, but, as Vogel believes, likewise, very essentially in their causes. Thus he is of opinion that serous dropsy owes its origin to a permeation of the serum of the blood through the walls of the veins; whilst fibrinous dropsy arises from a permeation of the liquor sanguinis through the walls of the capillary vessels.

The cause and mode of origin of serous dropsy are thus explained: "we are induced to believe that serous dropsy always proceeds from the venous system, and that it takes place as soon as there is a want of balance between the porosity of the venous walls and the specific gravity of the blood contained therein; that is to say, when the venous walls become more porous, or the blood lighter and more aqueous than in the normal condition. In either case there is an increased transudation of serum through the walls of the vessels. This is the manner in which local dropsy invariably occurs where individual veins are compressed, or, either for a time or permanently obstructed, as in cases of pressure from a tumour, or of complete obliteration. In this manner, the pressure of the impregnated uterus causes œdema of the feet; and pressure on the *vena portæ* and *vena cava ascendens*, arising from degeneration of the liver, or some other tumour, produce ascites and œdema of the lower part of the body."

Although the increased hydrostatic pressure of the blood is doubtless one of the determining causes of the effusion; yet, as the author observes, this phenomenon cannot, in the existing state of knowledge, be regarded as a purely mechanical process; nor does the above account explain all that takes place. How, for example, does it happen that the fibrin, which is dissolved in the plasma, remains in the vessels when the serum escapes; and, again, what is the reason why, as a general rule, these dropsical fluids contain as large an amount of salts, but more water and less albumen than

---

* "Neubildungen; literally, new formations."

the serum of the blood? No satisfactory explanation can at present be offered of these and similar phenomena; but it is most probable that they still depend upon some subtle chemical and physical conditions affecting the blood, and requiring only further and more successful investigation for their elucidation.

In speaking of *fibrinous dropsy*, the author appears not to be acquainted with the received opinions in this and other countries; for he says it is a species that has hitherto been seldom described, that its signification has not been properly interpreted, and that it has never been correctly distinguished from serous dropsy. It is somewhat surprising to find a pathologist of Dr. Vogel's attainments making these extraordinary statements, which the daily experience of all our readers must directly contradict. What is here termed "fibrinous dropsy," is that familiar form of effusion, partly of fibrine and partly of serum, which so repeatedly takes place, most commonly into the different serous membranes, but frequently in other situations, as in the parenchyma of organs. That this form of effusion "has never hitherto been correctly distinguished from serous dropsy," is a most absurd assertion. No practitioner would confound the two affections together; indeed, we are inclined to doubt whether the generality of medical men, by regarding effusion of fibrin as an inflammatory process and that of a serous fluid as the result of congestion, do not thereby establish the most important and valuable of all distinctions. The author gives only a more technical definition of the same thing in the following passage:—

"In serous dropsy, the causes of venous dilatation are frequently mechanical, and are, consequently, included in the department of pathological anatomy. Not so with fibrinous dropsy. Here the dilatation is dependant on dynamic causes, whose investigation would, of necessity, lead us far into the department of nervous pathology. * * * I restrict myself, at present, to the mere statement that fibrinous dropsy is essentially dependant on the capillary system; that it is associated with, and for the most part arises from, a dilatation of those vessels, and a tension and attenuation of their walls." P. 51.

This opinion, namely, that the capillaries are more especially the part of the vascular system concerned in fibrinous dropsy, is in all probability correct; for, although it is impossible to demonstrate the fact in the human body, the well-known phenomena connected with the dilatation of the capillary vessels, as determined by the microscopic examination of the lower animals, leave scarcely any room for doubt. But a similar difficulty to that noticed with respect to serous dropsy, where only the serum of the blood escapes, presents itself here:—

"Since the serous, and also the fibrinous fluids, take their origin from the blood, and are produced by the permeation of its fluid constituents through the walls of the vessels, how is it that in some cases we have one, and in others, the other form of effusion? In the present state of our knowledge, this question cannot be satisfactorily answered; there is, however, every probability that it admits of this solution: namely, that serous dropsy, as we have already stated, owes its origin to a permeation of the fluid of the blood through the walls of the veins, while fibrinous dropsy arises from a similar permeation through the walls of the capillary system." P. 50.

In connexion with this point, it must be borne in mind, what the above passage leaves in some doubt, though the fact is elsewhere noticed, that in fibrinous dropsy it is the whole of the liquor sanguinis that is effused, although subsequently, the serum often being absorbed, the lymph alone is met with. The general chemical character of the effused matter is thus described:—

"In its chemical composition, this fluid is identical with the plasma of the blood; that is to say, with the blood independently of its corpuscles: it is serum or the fluid of serous dropsy, with dissolved fibrin. * * * * The similarity of the fluid to the plasma of the blood occasionally extends even to their quantitative composition; usually, however, the dropsical fluid is the richer in water, and contains a less amount of organic constituents—albumen and fibrin. It is very seldom that this rule is reversed. In this point, therefore, there is the same relation as between the fluid of serous dropsy and the serum of the blood." P. 47.

It is well known that the chances of effecting an absorption of a fibrinous dropsy are much less than in the serous form of effusion. The probability of securing this important object is, however, remarkably dependent upon the situation and extent of the effusion, and, according to the author, upon the state in which the fibrin exists. As to this latter point, Dr. Vogel affirms that, as long as the fibrinous fluid is not coagulated, it may, like the serous fluid, be resorbed, partially or completely; and even, he thinks, with a greater facility, on account of there being no impediment to the activity of the venous system. "But if the fibrin be once coagulated, then the resorption can only extend to the serum;" unless, indeed, it can be rendered fluid again by chemical remedies, such as iodine, which, "for the present, remain unanswered questions." We cannot agree in the whole of these statements. As to the greater facility of absorption of liquid fibrin, as compared with serum, this must be mainly a matter of surmise; and, recollecting the rapid disappearance of ordinary dropsies, which occasionally takes place under active treatment, it does not seem to be a likely occurrence. Then, again, the author speaks doubtingly of a firmly established fact in pathology, the possibility, namely, of coagulated fibrin being absorbed. In iritis this is constantly seen, and the same thing is known to take place in effusions of solid fibrin occurring in the parenchyma of organs, as in the lungs.

Dr. Vogel notices an interesting fact, that when the dropsical fluid is enclosed in a sac of coagulated fibrin, and is in a manner thus cut off from the absorbing vessels, be they veins or lymphatics, the resorption is a more tedious and difficult process. We have lately met with a most striking example of a multitude of cysts, varying in size from a hazel-nut to an orange, formed of effused and coagulated fibrin, and containing serous fluid. The case was originally one of extensive chronic peritonitis affecting almost every part of the serous membrane, which was uniformly indurated, in some places cutting like a scirrhous substance, and also much thickened. There had been during life intense and long-continued suffering, with a feeling as if the bowels were bound together, and at other times a painful tearing sensation was experienced. Recent and most extensive acute peritonitis had supervened, producing the changes above noticed, and causing great swelling of the abdomen with a mixed feeling, on examination, of fluctuation and doughiness. Under the microscope, the immensely thickened peritoneum, exhibited scarcely any thing else than an infinite number of minute wavy fibres, like those of areolar tissue, and without a trace of the cells seen in true scirrhus.

As several notices of the Blood have recently appeared in this Journal, we pass by the Chapter on the pathological relations of that fluid, though it will well repay a careful perusal.

The larger portion of the present volume is occupied with what Dr. Day terms " Pathological Epigeneses." The original expression (*Neubildungen*) signifies literally " new formations." Although neither of these names is free from objection, we think, on the whole, that employed in the translation is the best of the two. It expresses, for example, more truly the relations of fibrous and fatty tumours to the normal structures ; these can hardly be regarded as entirely new productions, inasmuch as they resemble the natural tissues after which they are named, both when seen by the eye and when examined by the microscope. But the fact is that the error lies with the author, who in his zeal for philosophic generalization, has placed in one category, a number of essentially different morbid productions—pus, fibrous, fatty, and encysted tumours—tubercle, scirrhus and encephaloid—and even unorganized deposits, such as urinary and biliary calculi. It is clear that the laws regulating many of these growths and concretions are so totally distinct, that no benefit can accrue by regarding them, even remotely, from the same point of view.

After dividing pathological epigeneses into two groups, the organized and unorganized, Dr. Vogel observes that, like every thing else in Nature, there must be a matter from which they may be produced. To this, which may either be fluid or solid, he applies the general terms *plasma* or *formative matter*.

" A plasma may act as formative material either for organized or unorganized products, or for both at the same time. The plasma for unorganized formations, which is usually an aqueous solution, from which deposits are produced or crystals formed in accordance with chemical laws, we shall name a *mother-liquid;* the matter giving rise to organized formations which are chiefly produced by the formation of cells, we shall term a *cytoblastema,* or, for brevity, a *blastema ;* and lastly a plasma, from which organized and unorganized products are developed, will be designated as a *mixed plasma.*" P. 101.

As regards the plasma of organized pathological formations, or blastema, it must always be amorphous, that is to say, it must neither be crystalline nor have a definite organic form. It is evident that, if this principle can be firmly established, the old idea, namely, that a tissue may be directly converted into a pathological formation, must be overturned. This, for general pathology, is therefore a most important question, and is ably discussed in the work before us. We need scarcely remind our readers that the tendency in all sciences in proportion as they advance, is to refer their varied phenomena to a few general laws. In the particular instance of organization, this has been strikingly illustrated in late years ; and one of the most fundamental of these generalities is, that whatever may be the ultimate form of organized matter, whether normal or diseased, it has apparently but one origin, namely fibrine, which is therefore to be regarded as the plastic element in all nutritive processes. With respect to healthy nutrition, this position seems to be satisfactorily proved, at least there is at present no known example of albumen, fat, or other proximate principle, becoming directly organised. It may be thought indeed, and this is the opinion of Vogel, that, in the case of the egg, the prototype of all formative fluids, and which up to this time is not known to contain fibrin, there is an exception to this rule. But there is good reason to believe that the received opinions upon this subject are erroneous; for we ascertained some few years since, that the formative matter in the egg of the fowl lying immediately beneath the germinal membrane, and which is a compound formed by the admixture of the white and the yelk, differs from both those albuminous substances, by being perfectly liquid and not coagulable by the ordinary tests for albumen. This fluid matter is probably fibrin, and when it is recollected how similar is the chemical constitution of albumen and fibrin, and how readily the former is converted into the latter, of which the best example is furnished in the changes taking place in the chyle as it traverses the lacteal absorbents, there is no difficulty in conceiving of an analogous action going on in incubation.

Whatever doubts may yet remain upon the point just considered, there can scarcely be any with respect to the principle, that fibrine is essential to all morbid products: upon this question the author speaks most decidedly. After showing that non-azotized substances, such as fat, salts, &c. cannot act as blastema for organized formations, he proceeds to observe:

"Hence there remain, as the actual and potential constituents of the blastema, only the protein-compounds; although these are never found alone in the body, being always associated with the above-named substances. Further, all these protein-compounds are not susceptible of development. Fluids which merely contain dissolved albumen and the above substances never appear to act as cytoblastemata. In the common dropsical effusions, which are always rich in albumen, we never observe any organized products, unless fibrin be also present: this is at least the result of my own observations, which have been very numerous, and I am not acquainted with a single exception to the law. Moreover, fluids in which casein is the only protein-compound, cannot, as far as observation has yet shown us, act as blastemata. In the milk, for instance, as long as it contains merely casein, we never observe any pathological formations, for the granular bodies belong to the normal process of development of the milk; as soon, however, as any fibrin is present, morbid products, such as pus-corpuscles, may be formed in it. On the other hand, in all fluids which we regard as cytoblastemata for morbid products, fibrin has always been found: hence we must regard it as the necessary and apparently the most essential ingredient in the cytoblastema. This law respecting the necessity for the occurrence of fibrin in the cytoblastemata of morbid products, I have seen to hold good in several hundred cases, without a single exception." P. 107.

Having determined the first great principle connected with organized epigeneses, namely, that they all spring from fibrine, the organizable matter par eminence, Dr. Vogel enters upon another of these fundamental questions which lie at the bottom of all sound pathology—on what is the development of the cytoblastema dependent? He sets out with an assertion, for which we think there is no sufficient ground, to the effect that chemical action cannot be the efficient cause: the arguments for this opinion are, however, derived more from our ignorance than from our knowledge, and are, for this reason, not very satisfactory. A careful consideration of the phenomena connected with the coagulation of fibrine, and of the first and essential steps in the process of its organization, induce us to express an opinion that the changes perceived are rather of a physical than of a vital character. They are connected, for example, with an alteration in the form of matter, the liquid lymph becoming solid and fibrillated, phenomena indicating, like the conversion of water into ice or the formation of crystals from a saline solution, a subtle molecular action. The production of the peculiar corpuscles observed in coagulated fibrine, and evidently one of the earliest and most important steps in its organization, may be explained in a similar way. The predominating influence of chemical forces in the nutritive process, evinced especially in digestion, respiration, and secretion, of itself renders it a reasonable proposition, that the same agencies are operative in the whole of what is called the organic life.

Dismissing the question relating to the nature of the forces employed, we proceed with the author to inquire where they operate, and, to obtain what the Germans call a *standpunkt,* or starting-point, two hypotheses may be assumed:

"There are two different causes which may be supposed to affect the transition of the blastema in development; firstly, the cause may be grounded on the nature of the cytoblastema, and the formation may be developed with the same necessity which, under favourable conditions, compels the separation of certain crystals from their mother-liquid: or secondly, the transition in the development may be dependent on external conditions, independent of the cytoblastema, as for instance, on the influence of the surrounding parts of the body, &c." P. 110.

In order to ascertain which of these hypotheses is deserving of preference, it is necessary "to distinguish between the capacity of the cytoblastema in the progress of development (potentia) and the actual transition (actus)." With respect to the capacity, Vogel justly remarks, no one will deny that it essentially belongs to the cytoblastema; for, if it depended on external influences, then would every substance placed in similar relations undergo the same process of development, an assumption entirely at variance with experience. The author concludes, as regards the "actual transition," in the instance of morbid products, that, as a rule, it can only be effected by the agency of the surrounding parts. He admits, it is true, some isolated exceptions, such as that pathological elements, especially pus-globules, can be produced without the contact of organized tissues. The general inference, as maintained in the work before us, is then that, although the capacity for organization is inherent, in every instance, in the cytoblastema, the act itself is dependant upon the surrounding tissues, which are further believed by Dr. Vogel to have the power of converting the new matter into their own texture, although the nature of the blastema is not a matter of indifference, more particularly, as it would appear, in those diseases in which the morbid product is altogether different from the surrounding parts, as in scirrhus, encephaloid, &c. The views of the author on this interesting question are thus set forth:—

"In the process of regeneration and in hypertrophy, where the influence of the cystoblastema on the nature of the development is at its minimum, the development itself appears to be entirely dependent on the normal histological elements between which the blastema is effused. Thus, in regeneration and hypertrophy, the blastema between areolar tissue, becomes areolar tissue; in the vicinity of bone, it becomes cartilage and bone; between muscular fibres, it is converted into similar tissue;* at the extremities of divided nervous fibrils, it forms nervous substance, &c. The circumstance that these and no other structures are formed, cannot in these instances be dependent on the blastemata, which, as far as chemical analysis goes, seem to be the same; and it is entirely the influence of the surrounding parts that modifies the character of the development. Here then, if we may be allowed the expression, we are entering the department of *solid pathology.*

"But it may be further asked: it being granted that, in the above cases, the nature of the development is essentially dependent on parts of the body already formed, what is the case with those pathological epigeneses in which the resulting morbid product is perfectly different from the surrounding parts, as in scirrhus, encephaloid, tubercle or pus? Is not the abnormal character of the product dependent on a peculiar pre-existing blastema, so that there is always one kind of blastema for scirrhus, another for encephaloid, and so on?

"We are yet hardly in a condition to answer this question satisfactorily. It is quite possible that the elements of the peculiar structures of scirrhus and encephaloid may be traced to the blastemata from which they spring, and that, in accordance with the views of the humoral pathologists, the pseudo-plasma may be dependent on an abnormal chemical composition of the blood. Another explanation may be attempted which equally elucidates the appearance of these peculiar morbid products, namely, that the peculiarity of the epigenesis is not dependent on any property of the blastema, but on changes in the properties of the tissues influencing the blastema; and thus the explanation of these phenomena is again transferred from the department of *humoral* to that of *solid pathology,* or, since in many cases these changes are dependent on a change in the nervous influence, to that of *nervous pathology.* It is, however, in the highest degree probable, that in the majority of cases neither the one nor the other of these views alone is strictly correct, that for the most part changes in the cystoblastema and changes in the physiological properties of the tissues are conjointly at work in producing an abnormal epigenesis." P. 114.

In supporting his doctrine, Dr. Vogel takes as a basis the process of natural development as it is witnessed in the egg of the bird. Although entirely agreeing in the necessity of this mode of procedure, we cannot coincide with this distinguished pathologist in some of his deductions. Being satisfied that, in all speculations of this character, the ultimate facts of organization can only be discovered by setting off with the fixed assurance that simplicity and unity pervade all the works of Nature, we cannot admit that there is any essential difference, which the author affirms there is, between the nutrition of the perfect organism and the formation of the new being in the egg. In the latter case, it is certain and admitted, that the cytoblastema has not only the "capacity" of becoming developed, but also the power of actually effecting development; and the same energies must be attributed to the cytoblastema of the perfectly-formed animal. If this be granted, and it is a position which no philosophic anatomist would be inclined to doubt, it follows that, in the first class of epigeneses, or those in which, to borrow the author's words, "the epigenesis takes place in a manner perfectly analogous to that which occurs in healthy nutrition," so far from the influence of the cystoblastema

---

* This is erroneous; a divided muscle is united by a cicatrix of dense areolar tissue.

being, as asserted, at it minimum, it is in reality the efficient cause of the whole phenomena. As regards the second class, comprising scirrhus, encephaloid, tubercle, &c., so far, again, from there being any evidence that the surrounding tissues are the determining cause of the development of the cytoblastema, it is seen that the latter, when it has undergone its morbid metamorphosis, has, in the first place, no resemblance whatever to the tissue around, which, in accordance with the theory, it ought to have; and in the second place that, whatever may be the tissue in which the deposit takes place, whether in the areolar tissue, in a gland, a muscle, or a bone, the new matter always presents the characters proper to its peculiar nature. Tubercle, for example, always offers the same aspect and microscopic appearances wherever it is examined, in the brain, in the lungs, or in the testis.

Although, at the first blush, it may seem that the hypothesis which attributes the act of development to the surrounding parts, especially in the first class of epigenesis, where it is said, in obedience to what the author proposes to call " the law of analogous formation," that bone forms bone, and muscle produces muscle, is a probable explanation; yet, when closely scrutinized, it is beset with difficulties. If the blood-vessels are supposed to be the controlling agents, what notion can we form of their modus operandi? The actions of these organs have been very much curtailed by the exact anatomy of the present day: and under no reasonable supposition can they be admitted to act beyond their defined limits, in the manner in which they must operate, if they be the formative agents. Then with respect to the nerves, it is positively known that their power is not essential to nutrition; a considerable part of the chick, for instance, is formed before the nervous system appears; and in plants there are epigeneses, but there are no nerves. If, in answer to the opinions we have advocated, it be said that it cannot be understood how it happens that from one of the same substance, fibrine, such a multitude of different products arise; a sufficient reply is furnished by the fact, that tissues as diverse from each other are in the chick, in obedience to the mysterious laws of organization, formed from and by that one material or cytoblastema lying beneath the area germinativa. It is, however, probable, as indeed Vogel himself surmises, that the cytoblastema is not always the same form of fibrine; and that in these modifications may be found one of the causes leading to so many heterogeneous productions.

In considering individual new formations, the author premises some general remarks on the cell-theory of Schwann, in the truth of which, as applicable to morbid productions, he on the whole coincides; but, like other observers, he finds it necessary to restrict or to qualify the general position that all tissues spring from nucleated cells. Thus, in scrofulous and typhous exudations, and in a great part of the cases of tubercle, there is at first a finely granular or even an amorphous matter, which by degrees breaks up into a more or less fluid magma, with irregular molecules, but no true cells. As this subject has on several occasions been considered in this Review, it will suffice to extract the following paragraph, in which the general views of Dr. Vogel are stated:—

" That this mode of development from cells occurs in pathological epigeneses may be readily shown in numerous cases. This process can be most obviously traced in the formation of pus-corpuscles, when they are produced from a fluid blastema on a free surface, or in a cavity connected with the exterior of the body. In such a case, we first observe numerous isolated granules, which become surrounded by a very delicate transparent cell-membrane, which subsequently forms so thick and opaque a wall, that the nucleus can no longer be seen through it; on the addition of acetic acid, which dissolves the cell-wall, or at any rate renders it transparent, the nuclei again become visible. If it is impossible to trace the whole course of development in one and the same cell, we can yet make out the successive changes of the whole mass of cells with sufficient certainty. These and similar observations, such as for instance may be made on the formation of epithelium, confirm the opinion that in all essential points Schwann's theory is applicable to morbid formations; but that, in individual cases, many facts may be observed which do not coincide with this theory, or at least render some modification imperative." P. 118.

The 5th Chapter treats of the special characters of pathological epigeneses, both fluid and solid, and, from the numerous subjects investigated, occupies the larger portion of the present volume. The section relating to purulent matter does not contain anything so novel or important as to require notice, nor indeed do several of those which succeed, concerning the epigenesis of areolar tissue, of blood-vessels, &c.

In speaking of melanotic productions, it is stated that, although the black matter is sometimes contained, as in the case of normal pigment, in true cells, usually however irregular in form, it frequently happens that these are wanting, the pigment molecules being deposited in the parenchyma of the affected organ. Although this description corresponds with what is seen in a microscopic inspection, yet it is questionable whether the pigment is ever deposited originally except in cells; at least the well-ascertained phenomenon of secretion in other tissues is opposed to any other conclusion. The scattered molecules seen in their sections may be accounted for by the rupture and disintegration of pre-existing cells.

The former history of facial neuralgia, in which, after the operation of dividing the affected nerve, the pain often returned, sufficiently indicated that the nervous tissue was capable of reproduction; an inference since substantiated by the observations of Nasse and others, who have found that, after division, and indeed when small portions were excised the fibres of nerves become re-formed in part, but they are somewhat smaller in size and fewer in number than in the normal state.

The attempts hitherto made to classify tumours have not been very successful; we do not, however, for that reason despair of an object, so important to external pathology, being accomplished. There is a very obvious cause of failure in the fact that, in order to determine the essential characters of tumours, microscopic observation is one of the principal means involved; and we need not remark that this mode of investigation is of very recent application, especially in this country. That it may often be used most advantageously in practice, we had recently a very conclusive proof, in a case of subcutaneous tumour removed in one of the metropolitan hospitals. The operator, from the suspicious appearances of a part of the diseased mass, and from its indurated character, was apprehensive that it was of a malignant nature, though another distinguished surgeon thought it was merely a modification of lipoma. To decide this somewhat doubtful point, a section of the above part was submitted to the microscope, when the whole was found to consist of two normal tissues, the adipose and the areolar, and the fears of malignancy were satisfactorily removed. Another instance occurs to us of a supposed scirrhous disease of the testis, which was clearly proved, by minute examination, to be nothing more than a deposit of scrofulous matter. Now, as it is one of the highest privileges of our science to solace human suffering, an unerring test, and this in many instances microscopic inspection affords, which will enable the surgeon, not by a mere guess unsatisfactory to himself, but with an assured conviction of the truth of his diagnosis, to inform the patient and his friends that a previously doubtful tumour is not malignant, would be to a conscientious practitioner a thing of great price. For these reasons we do not concur with the opinion of the author, that "all attempts to arrange tumours (as we do animals and plants) into genera and species, must necessarily fail." On the contrary, the very attempts made in the present and other works, and which, imperfect as they still are admitted to be, are of primary importance in practice, pre-suppose that there is a natural system, as in all branches of science.

The classification of Dr. Vogel has nothing of novelty in it, as our readers will gather from the subjoined extract.

"In a histological point of view, tumours may be arranged in two great divisions. To the first belong those whose elements may be considered histologically to agree with those of the normal body, and which further being once formed, discharge the duties of the normal constituents of the body, take a part in the general metamorphosis of tissue, and are nourished and increased like other parts. These are homologous, non-malignant tumours.

"In the second division, we must place those whose elements in a histological point of view differ more or less from those of the normal body, and which (as in the process of suppuration) from their nature give way, soften, and destroy the organic parts which surround them or which they enclose—heterologous, malignant tumours." P. 203.

In the account of the individual groups many interesting details are given, which we regret our limits prevent us noticing, excepting in a cursory manner. The great obscurity in which the tumours of bone are involved, renders all judicious attempts at simplification and distinctness acceptable. In this respect the several sections treating of fibrous, cartilaginous, and osseous tumours, may be studied with advantage. Those consisting of cartilage have been divided, since the excellent researches of Professor Müller, into two groups; the first consisting of true cartilaginous tumours, usually appear as hypertrophy and abnormal growth of bone (as callus, exostosis, &c.), and gradually pass into the osseous tissue; the second, comprise, distinguished by the term *enchondromata*, on the contrary, do not ossify, although they occasionally offer some ramifying corpuscles suggestive of the well-known osseous lacunæ or bone-cells. Enchondroma appears under three distinct forms; two of which occur in the bones, and a third, much more rarely, in soft parts, especially in glandular organs, as the parotid, testis, and mamma.

"It forms a roundish, and generally smooth tumour of variable size, which on a section being made allows even the unaided eye to recognize two distinct constituents, one fibro-membranous, and the other gray, transparent, and soft, resembling firm jelly or softened cartilage. The latter element shows under the microscope roundish or elliptic cells varying from the 150th to the 50th of a line in diameter, and sometimes even larger, which enclose a granular nucleus ranging from the 200th to the 300th of a line in diameter." P. 225.

These tumours are not malignant, are unaccompanied by pain, and are so slow in their development, that they may exist and increase for ten or twenty years, attaining a considerable size without materially incommoding the patient.

"Gluge describes a tumour of this kind, which, on extirpation weighed nine pounds and a half. They may, however, when large, like the non-malignant tumours formerly described, inflame and ulcerate, and become dangerous from the quantity of the discharge." P. 228.

The central enchondroma is developed in the interior of bones, and is the most frequent of all; it usually appears in the metacarpal and metatarsal bones and in the phalanges, as rounded smooth tumours of variable size, encased in a vesicular, expanded, osseous cortex, which is at some points absent, as if the tumour had burst through its wall. Peripheral enchondroma originates on the surface of the bone, and has therefore no bony sheath, being covered only by periosteum. Its form is more irregular than the preceding, and its surface is rendered rugged and uneven by the separate rounded cartilaginous formations which protrude as distinct nodules, varying from the size of a pea to that of a cherry: this form is principally observed in flat bones, namely, the ribs, and cranial and pelvic bones, rarely in the cylindrical class. These tumours were formerly confounded with many others occurring in bones, under the various names of spina ventosa, osteo-sarcoma, osteo-steatoma, &c. (p. 229.)

Under the head of "Encysted Tumours," Dr. Vogel has collected most of the later observations of different pathologists. His definition of this important class is thus given:—

"The true, simple encysted tumours not only possess a perfectly closed membranous sac, but it is also essential to their character, that the contents of this sac are either not at all, or only very imperfectly organized, and show no organic connection with the sac itself. This forms the distinction between encysted tumours and the enclosed fatty and fibrous tumours formerly described, in which the envelope, consisting of areolar tissue, throws out organized elongations, and processes not only into the substance of the surrounding parts, but even into that of the tumour itself; thus not so much separating the tumour from the surrounding parts, as forming a medium of connection between them." P. 239.

There is another class, which the author calls, with Müller, cyst-like tumours (cystoid).

The true encysted tumours may be grouped under two tolerably well characterized subdivisions. The first class embraces those which contain aqueous or serous contents, and of which three forms are described: one, of which the hydrocele of the cord, the cysts of the choroid plexus, and especially the cyst of the parenchyma of the ovaries, are examples, is in fact a circumscribed local dropsy, the fluid being effused in a part consisting of lax areolar tissue or under a thin membrane (as beneath a serous membrane): another kind, originates simply in an obstruction of the excretory duct of a secreting organ; it is often seen in the kidney: a third variety, more deserving the name of encysted tumours than the two preceding, consist of a perfectly closed cyst, which externally is firmly connected with the surrounding parts, but internally exhibits a smooth surface, which, in the progress of formation becomes invested with an epithelium.

"The second division of the simple encysted tumours is distinguished from serous cysts by the circumstance that the contents do not consist of an aqueous fluid, but contain peculiar corpuscular particles which render them thick and pulpy. They sometimes resemble honey, sometimes boiled groats, and occasionally they have a gelatinous appearance. In accordance with these varieties in the contents, these tumours have received different names, and been termed hygroma, meliceris, atheroma, gummy tumour, &c. Such names are, however, in the highest degree, vague and unscientific." P. 245.

The inner surface of the cyst is furnished with a decided epithelium, and the cells of this, in a state of successive formation, are continually being thrown off and so form a principal part of the contents, as in the case of the tumours connected with the sebaceous follicles, of which an interesting account has been given lately by Mr. Erasmus Wilson, and other writers. In addition to these cells, fatty matters of various kinds (olein, margarin, cholesterin) are almost invariably present; various extractive matters and salts also form a part of the contents. If the calcareous salts (phosphate and carbonate of lime) are deposited in considerable quantity, the cyst, as well as its contents, becomes entirely or in part converted into a concretion, or, as it is usually expressed, the encysted tumour appears ossified.

The author next describes those remarkable contents of encysted tumours, consisting of masses of hair, bone, teeth, &c.; but, as these are well known, it is not necessary to dwell longer on this subject.

The study of malignant epigeneses or *pseudoplasmata* is attended with more difficulty than even that of homologous tumours. Thus at present some of the primary conditions, essential equally for forming correct generalizations and for arriving at details, are wanting. For example, the true organic relations existing between the normal tissues of the body and malignant productions, are not known; so that it is still a questionable point with some practitioners, whether a tumour, originally and essentially non-malignant, can become malignant in the course of time, from internal constitutional causes, or from external accidental causes, such as a blow. Again, how vaguely are many terms relating to these growths, such as "tubercle," applied; then what uncertainty exists among the generality of medical men, as to the vascularity or non-vascularity of tuberculous matter. It is fortunately true that much of this uncertainty is beginning to disappear before the accurate and extended researches of late years, made with reference to scirrhus, tubercle, &c.; and there can be no doubt that a persistence in the same course will ultimately substitute clear and exact knowledge for the existing confusion and uncertainty. Especially we may hope for the most valuable results, from the sedulous attention that is now paid to morbid states of the blood and nutritious fluids; for the history of these malignant epigeneses, and particularly of tubercular disease, indicates that they are intimately connected with a depraved condition of the circulating fluid. How otherwise can we account for the production of scrofula and phthisis, which are known to arise from habitual residence in a vitiated atmosphere? This inquiry acquires a higher interest than attaches to the discovery of the primary causes of other diseases, both because it promises to lead to more immediate benefits in the way of prevention, and because it relates to a class of affections which, when once established, are at present almost or entirely uncontrollable. Past experience shows in a marked degree, that we are here to look to preventive rather than to curative means for success; a consideration which should induce the medical practitioner to direct increased attention to an improved hygiène.

The distinctive marks of malignant tumours, when contrasted with the non-malignant, are thus given:—

"Malignant tumours proceed of necessity to softening from esoteric causes; the softening being a necessary consequence of their development.

"This circumstance sufficiently distinguishes the two classes of tumours. But other pathological formations also soften without being, on this account, malignant. Thus, for instance, softening takes place in all suppurations in which the pus is developed from a solid cytoblastema. In this case, however, the morbid epigenesis alone softens, the original tissues taking no part in it; if the pus is evacuated externally or becomes resorbed, they return to their original condition, and re-assume their previous functions; the affected part, with the exception of some trivial changes which occasionally remain, is restored *in integrum.* The case is far different with malignant tumours. In these the softening is not confined to the morbid epigenesis amongst the original histological elements, but the latter become themselves involved in the process of softening, and are also destroyed, so that the expulsion of the mass from its place of formation is attended with a loss of substance. The softening of the pseudoplasmata, therefore, is not innoxious, but malignant and ulcerative; it consists not in a healthy suppuration, but in a process of ulceration.

"The difference between non-malignant and malignant softening is not confined to the above points; it extends even to the morphological formation of the product of the softening. In the non-malignant softening this consists of the normal pus-corpuscles formerly described; in the malignant, on the other hand, of very irregular molecules, which show scarcely a trace of organization, and resemble the products of the putrefaction of organic bodies, mixed with fragments of the destroyed tissues." P. 262.

The softening usually commences not upon the surface, but in the interior; and, as the product of this change is not therefore immediately discharged, "the ichorous pus continues for some time in contact with the walls of the vessels, and, by endosmosis, its fluid parts are taken up into the lymph and blood, exerting a morbific influence, in a manner at present but little understood, upon these fluids, and thus gradually inducing a general cachexia." The author, entertaining these views, attaches a high importance to the removal of the malignant tumour *prior to softening*. He says, the evil consequences just noticed "may be obviated by the removal of the pseudoplasma by operation previous to its softening, and upon this rests the advantage to be derived from the employment of the knife in the malignant epigeneses. In order to be useful, such an operation must be radical, *i. e.* no part of the pseudoplasma should be left behind."

Dr. Vogel subsequently adds, that although tumours absolutely corresponding in all other points with the malignant, may, as in the case of pulmonary tubercle, cause merely local destruction and subsequently heal, or, as in some scirrhous growths, be removed by surgical operation without recurring, yet these are the exceptions; for "there commonly arise on other spots of the body, either in the vicinity of the original pseudoplasma, or distant from it, simultaneously with the first, or subsequently, other pseudoplasmata of the same kind."

With respect to the classification, the author observes :—

"The malignant epigeneses are as little capable of precise classification as the non-malignant tumours. They may, however, according to the higher or lower degree of organization which they attain before their disintegration, be reduced into certain groups, but these are still less strictly separable than in the case of the non-malignant formations; transitions between the individual forms are of common occurrence, and, indeed, the same tumour not unfrequently shows totally dissimilar elements; the reduction of these formations into numerous species with a multiplicity of names is, therefore, quite unjustifiable. We discriminate, firstly, pseudoplasmata which are slightly or not at all organised; and secondly, such as attain a higher grade of organization. As representatives of the former class may be pointed out the depositions in typhus, and in scrophulous tumours; of the second, encephaloid and scirrhus. Many varieties of tubercle form a connecting link between these leading divisions." P. 259.

If by organization Dr. Vogel indicates vascularity, we must dissent from the last paragraph, as, from repeated observation, we are satisfied that the true scrofulous and pulmonary tubercles are non-vascular.

As the matter of typhous deposits has not attracted so much attention as other malignant formations, we extract the following particulars respecting it. The most common seat of this epigenesis, although it may occur in different parts of the body, is, as it is generally known, in the intestinal canal between the mucous and the muscular coat, in Peyer's glands (especially at the termination of the small intestines), and in the mesenteric glands; less frequently in the spleen and lungs, and in and under the mucous membrane of the trachea.

"These formations usually appear as a more or less firm lardaceous mass of a yellowish or whitish colour, which is deposited in greater or less abundance, amongst the normal tissues, gradually softens, and, as the normal elements of the region become also involved in this process, forms ulcers, which either heal by cicatrization, or continue until the death of the patient. In many cases death takes place before the commencement of softening." P. 272.

There can be no doubt, from the manner in which it fills up all the interstices of the tissues, which are, as it were, infiltrated, that the matter is deposited in the fluid state and subsequently assumes the solid form by coagulation; the author has never seen an instance in which it remained liquid.

By a microscopic examination, the following constituents are recognized in the mass :—

"1. An amorphous, semi-transparent stroma.

"2. Molecular granules from a size too minute to estimate, to the 800th of a line in diameter; sometimes interspersed with larger fat globules.

"3. Larger corpuscles (imperfect cells and cytoblasts) from the 800th to the 300th of a line in diameter, rarely larger. Some of these enclose smaller corpuscles (elementary granules and nucleoli) which are wanting in others." P. 272.

The softening of typhous matter usually proceeds rapidly, following the deposition in the course of a week or less. It is an interesting observation, as indicating an affinity in the results as well as the causes of typhus fever and scrofula, that "the typhous matter cannot be histologically distinguished from the deposits which occur in scrofulosis and tuberculosis."

With respect to the chemical character of this morbid production, Dr. Vogel conceives it to be chiefly composed of the fibrin of the blood, pervaded, however, as in all similar cases, by other elements of that fluid, and poured out in a fluid state from the capillary vessels.

"The question here suggests itself: Is this fibrin normal, or has it, whilst still in the blood, undergone a specific change? The possibility of such a change cannot be denied, since we know that fibrin is very transmutable: but the assumption of such a change without a demonstration of its nature by organic chemistry, is of no advantage in a scientific point of view." P. 274.

After stating the possibility that the diminished energies of the original tissues may also be assumed as a probable cause why the exudation does not become organized, the author concludes by saying,

"With this brief view of the subject, I wish to express myself as opposed to the opinion, that there exists in the blood a specific typhous matter, with the deposition of which, in certain parts of the body, the disease localizes itself and terminates. At the same time the local importance of this deposit cannot be questioned. A great number of cases of typhus proceed to a fatal termination from the effects of these depositions, from ulceration, perforation of the intestine, &c." P. 274.

The second sub-class of pseudoplasmata slightly or not at all organized, are scrofulous deposits, of which *tubercle* is the most important and the most frequent form. Dr. Vogel states, as we have seen above, that scrofulous matter cannot be with certainty distinguished histologically from typhoid or tubercular matter; but the whole process of deposition and softening is characterised when contrasted with that of typhus by its extreme slowness, occupying in the one case as many weeks or even months as days in the other case. In defining the signification that ought to be attached to the vaguely applied term "tubercle," the author thus expresses himself:—

"Originally the name 'tubercle' was a very general one; in accordance with its proper signification, it expressed all nodular tumours, and even at the commencement of the present century, Baillie applied the term to fibrous tumours of the uterus. At the present day, however, its meaning is much more limited, and by tubercles we now understand those pathological epigeneses which are engendered in consequence of a specific disease or morbid tendency—*tuberculosis.*" P. 276.

This is a restriction perfectly essential to a correct knowledge of this common and formidable deposit.

As in the case of typhous matter, there can be no doubt that tubercular deposit passes out of the capillary vessels in a liquid form. The most favourable situation to watch the whole process is in the air-cells of the lungs; but to arrive at certain results perfectly successful injected specimens, in all stages of the disease, are indispensably necessary; and these, unfortunately, are obtained with difficulty. The following are the results of our own observations made on such preparations:—When the deposit is commencing, and when it is in such minute quantity as to escape the naked eye, the tubercular matter is seen as very small whitish masses, occupying distinctly a part or the whole of single air-cells, and presenting a resemblance to minute drops of white wax. There can be, as we have already said, no uncertainty as to its original fluid state; but we entirely agree with Vogel in asserting, that no one has seen it in that condition, for the most minute particles recognisable by the microscope are solid. As the deposit increases, and as contiguous air-cells become filled up, the vascular retia on their walls are gradually compressed and ultimately absorbed; and thus the previously isolated tubercular masses blend together, and form the larger accumulations seen by the naked eye, a process which was first distinctly traced and described by Mr. Rainey. There is no reason to suppose that the phenomena are different in other textures, though of course they must be modified by the local circumstances. The account given by the author in the following passage, differs in some important points from what is stated above, and is, we are satisfied, erroneous in affirming that the normal tissues are usually neither displaced nor altered; we refer here, to such parts as blood-vessels, &c., for it is true that the elastic pulmonary membrane, in the particular case of the lung, is not entirely, if at all, destroyed.

"Whenever tubercles are observed in what may be presumed to be their earliest stages, they appear solid, form a more or less dense mass, and fill up all the interstices of the elementary tissues in which they are deposited. The tissues are usually neither displaced nor altered by the tubercular matter; on the contrary, they in general retain their normal position; they are, however, as closely and perfectly invested by it, as the stones of a wall by the solidified mortar which has been applied between them. We can most readily convince ourselves of this condition by treating fine sections of tubercular deposit from the lung with acetic acid or caustic ammonia. By means of these reagents the opaque tubercular matter is rendered transparent, and under the microscope, the enclosed portions of lung (the intersecting fibres) are perceived to be arranged amongst the tubercular matter just as in the normal state." P. 278.

The microscopical characters of tubercle have been so often described that it is not necessary again to refer to them. The author, in noticing the numerous and conflicting opinions of writers respecting the chemical composition, says that nothing is at present certainly known, beyond the fact ascertained by Lehmann and repeatedly confirmed by himself, that tubercular matter consists principally of a protein-compound.

In the second great class of malignant formations, Dr. Vogel includes the various forms of carcinoma—encephaloid (cellular cancer), scirrhus (fibrous cancer), melanotic (or a combination of melanosis and cancer), and colloid (gelatinous cancer). One of the leading principles connected with the whole class is, that all cancerous affections consist of a new formation, totally distinct from the normal tissues, but mixed up with a larger or smaller proportion of the original healthy structures; so that few, if any, cases of cancer are altogether malignant as to the constitution of the tumour.

With respect to the individual varieties of the disease, they vary among themselves mainly according to the relative preponderance either of the new material or of the normal texture. The microscopical examination of carcinomatous growths, seems to have thrown a more clear light upon the causes of these differences, by showing that, whereas the purely cancerous matter consists essentially of *cells*, variously modified and mixed with a peculiar viscid fluid; the normal tissues retain their characteristic marks, and may thus be still recognised. That, in scirrhus and other similar affections, a considerable portion of the tumour is often made up either of the original structures of the body, or of fibrin effused by common inflammatory action, has long been known: and herein is to be found the explanation of those cases, which from time to time are adduced as proofs of the possibility of curing these hitherto unmanageable diseases; the fact being, that the absorbent action is, in these instances, excited, and the healthy tissues, or the inflammatory deposits being removed, it is erroneously affirmed that the special and essential epigenesis is eradicated. It is not necessary to give the author's account of the microscopic elements of cancer, as it does not contain any novel information. The cancerous matter, he believes, as in the case of tubercle, is effused in a liquid form, and so becomes infiltrated into the tissue of the affected part, undergoing subsequently a process of coagulation.

"The cancerous matter occurs between original elementary parts of the parent tissue, and occupies, more or less perfectly, all the interstices. A slight infiltration of cancer in a tissue, frequently escapes the observation of the unaided eye, and can only be detected by careful microscopic examination—as, for instance, in fatty tissues. When the interstices are not thoroughly filled, and the cancerous deposit is soft, the parent-tissue, at least in the first stage, is comparatively little injured. If, on the other hand, the infiltration is complete and the cancerous deposit very firm and solid, then the elements of the tissue become compressed, appearing to be blended with the deposit into a homogeneous mass, and gradually become atrophied and disappear. This disappearance of the elements of the tissues by atrophy and resorption, which is peculiar to the first stage of cancer, previous to softening, must be clearly distinguished from the destruction of the entangled tissue, which is dependent on the softening of cancer, and of which we shall speak presently." P. 301-2.

The vascularity of carcinomatous tumours varies, as it is well known, in different forms of the affection; in some cases, blood-vessels are almost or entirely absent; whilst in other instances, and especially in the soft varieties, as fungus hæmatodes, they are so numerous that, when ulceration has taken place, the exposed surface bleeds on the slightest contact.

The general views of the author, as to the nature and progress of these affections, may be gathered from the following extract, with which we must close our notice of this subject:—

"Simultaneously with the process of development the cancerous tumour undergoes other changes; it increases to such a degree, that from a very limited origin it often becomes distributed over a large space, occupying one or even several organs. This enlargement is undoubtedly dependent on the cellular structure of the cancer, and probably also on the fibres acting upon the nutrient fluid in the neighbouring parts, in accordance with the law of analogous formation. The increase of the cancerous cells is forwarded by the circumstance that many of them act the part of parent-cells, and contain in their interior young cells, which in all probability are capable of a similar mode of increase. Moreover, the numerous cytoblasts frequently observed in a cell, probably all become themselves developed into distinct cells. With these facts before us, there is clearly no limit to the increase of cancer-cells, neither is there any necessity for regarding them as distinct organisms similar to the lowest fungi and algæ. It is clear, however, that the fibres and the vessels (if any are present) cannot be increased by means of the cancer-cells; in all probability the increase of the fibres—and in fibrous cancer such an increase undoubtedly occurs—is dependent upon the influence of the pre-existing fibres, just as is the case in the growth of pure fibrous tumours. The innate capacity for augmentation possessed by cancer, is very energetic, and forms an essential distinction between cancerous tumours and scrofulous depositions; for, in the latter, this capacity is either altogether absent, or only present to a very slight degree. Hence the growth of cancer is most rapid when an increased cytoblastema is yielded to it from any source, as, for instance, from inflammatory exudation, especially from fibrinous dropsy in the adjacent parts. It always increases on the supervention of softening and ichorous discharge, in consequence of the irritation to which these processes give rise in the surrounding parts. The exudation thus yielded by the neighbouring hyperæmic parts is converted into cancerous matter, and hence cancer is not, as is frequently the case with tubercle, separated from the surrounding parts by granular cells or pus, nor is it retarded in its growth by a line of demarcation. The newly-formed cancerous matter goes through precisely the same course of development as the original; proceeding of necessity to softening. In some cases, we find the peripheral portion of the cancerous matter, and the surrounding parts contending, as it were, for the cytoblastema, and sharing it between them. There are formed, as may be observed in cancerous ulceration, fungoid and extremely vascular granulations; but these are always so infiltrated with cancerous matter, that, after a very brief existence, they soften and become disintegrated, never contributing to the formation of persistent tissues." P. 304-5-6.

In the 8th Chapter, Dr. Vogel gives a brief but interesting summary of the parasites, vegetable and animal, or, as he terms them, "independent organisms," which infest the human body—a subject of considerable importance in practical medicine, and which has, in late years, attracted a large share of the attention of the most eminent zoologists. In many instances, it is no easy matter to account for, or even to conceive of, the origin of entozoa, and their entrance into the organs where they are met with; more especially if we adopt, as we are inclined to do, the definition of Vogel, who limits "the acceptation of parasites to those organic structures whose germs have penetrated into the organism *from without.*"

In the case of the entozoa, which have their habitat either in those cavities of the body that have a direct external communication, such as the various intestinal worms; or which are domiciled in organs having an indirect connexion with the exterior, as the distoma hepaticum in the gall-bladder, and even the formidable strongylus gigas lodged in the kidney; in these and similar cases, although the origin from without may not be very obvious, still it may be comprehended. But what is to be said of those parasites which exist in the parenchyma of solid organs, or in cavities closed in on every side. How, for instance, does the trichina spiralis find its way into the very sheath (sarcolemma) of the primary muscular fasciculus, or the cysticercus into the brain or chamber of the eye? The difficulty of explaining these phenomena by the ordinary mode of reproduction, has led to the idea of spontaneous generation; a doctrine which, although from time to time still advocated, is opposed to every real advance in the science of embryology. In the following extract the author, after setting forth the arguments that have been adduced in favour of this theory, thus opposes them.

"Let us now consult experience for materials in order to reply to this question. We find that in all cases where opportunity has been afforded of tracing, by direct observation, the origin of an organism, it has taken place by propagation; whilst, on the contrary, not a solitary unexceptionable observation of a spontaneous origin exists in the records of natural history. Analogy is, therefore, completely in favour of the view that propagation is the only manner in which existing organisms are engendered. * * * * The objections which have been urged against this view, and the arguments which have been adduced in favour of a spontaneous production of parasites, rest chiefly on the ground that in many cases the origin of these organisms, by means of propagation, is inexplicable; and is, therefore, held to be impossible. But it is overlooked that the assumption of their spontaneous origin is in reality merely a formal explanation, which leaves us completely in the dark respecting the true reasons and conditions of their production. Moreover, many of these reasons have latterly become invalidated by the progress of knowledge, since not merely the possibility, but also the reality of their propagation to other organisms, and the inducing conditions, have been demonstrated in various parasites; and although in this respect at present much appears mysterious, yet the numerous experiences of latter years must raise a hope in every unbiassed observer, that the further advancement of knowledge will clear up the obscurity which at present envelops this province, and will establish the origin of all parasites by propagation, to the exclusion of spontaneous origin." P. 424.

It follows, from this view of the question, that parasites are never a true product of disease, and cannot therefore originate from degenerated particles of the body, depraved secretions, &c.: a position which does not, however, invalidate the commonly-received and firmly-established opinion, that certain morbid changes of the body favour the development of entozoa by furnishing those conditions under which alone these animals can exist.

"Thus, for example, vegetable parasites (fungi) do not in general develop themselves upon mucous membranes, until, by morbid processes, a deposit of coagulated fibrin, which serves as a bed, has become prepared for them, and until this exudation has passed into a state of putrid decomposition. An abundant secretion of mucus favours the development of worms which have entered the intestinal canal from without. Some states of the organism, on the contrary, disqualify it as a habitation for parasites. Thus, most of the entozoa in the intestinal canal are expelled by increased peristaltic action; some fluids of the body, as bile, urine, gastric juice, and some medicines, prove deleterious, and indeed fatal, to some of them; inflammation, or at least suppuration, may injure, and even destroy them." P. 425.

A part of the mystery still involving this inquiry has been cleared away by the admirable researches of Steenstrup, which were noticed in a former number of this Journal (see Med.-Chir. Rev., July 1846, p. 22). The leading fact therein demonstrated, that there are, namely, in many classes, and especially among the entozoa, animals which act as nurses, giving birth to new beings without being truly prolific, elucidates many points in the reproductive process of parasites. The generation of the guinea-worm (*filaria medinensis*) is so remarkable as to have given rise to some doubt as to its real character: "they are viviparous, and contain in their interior such a prodigious quantity of young that some maintain the worm is not an animal at all, but a membranous sheath filled with small worms." It is further peculiar, although the existence of a male is known, that as yet only female filariæ have been found in the human body. It may then be asked—do females alone, while still young but after their impregnation, enter the body, because they there find conditions favourable to their further development? (*L. c.*, p. 457.) All these circumstances seem to indicate a resemblance to some of the stages described and depicted by Steenstrup; but it is proper to remark that the nematoidea have not presented to that careful observer any distinct evidence of alternate generations, so that further information is required upon this interesting question.

Among the parasitic animals one of the most curious is the *acarus folliculorum*, belonging to the arachnida, discovered by Simon of Berlin and since described by other observers. It is sometimes spoken of as occurring so frequently that its presence can scarcely be regarded as abnormal. We believe, however, that there is some mistake in this statement: at all events it is no easy matter to procure specimens for the microscope, a circumstance difficult to reconcile with some of the accounts that have been published. According to the author, the a. folliculorum very frequently exists in the hair glands of the human subject, on the nose, upper lip, and the glands of the beard, being sometimes solitary, whilst at other times ten or more are found in a single gland.

There is no subject on which the comprehensive and accurate researches of modern anatomy have thrown so much light, as that relating to malformations. The older observers, troubling themselves but little with the general laws of organization, rested satisfied, like their brethren of the geological school, with regarding all deviations from the ordinary course of things as lusus naturæ. Speaking of these *vitia primæ conformationis*, Bischoff, in his interesting sketch,* says justly, "for a long time they were rather objects of affright and aversion, of superstition and of curiosity, than of scientific investigation." It is, however, certain that by far the greater number of congenital malformations are susceptible of explanation according to the known principles of development and of nutrition; and, when closely scrutinized, notwithstanding the wide departure from the normal process they may seem to present, they fall into definite classes recognizable by the embryologist as belonging to certain phases in the development of the fœtus. So much, indeed, is this the case, that the study of malformations and of developmental anatomy mutually illustrate each other; and it is not too much to affirm that the former, as a philosophical investigation, could never have been successfully cultivated without the aid and the elucidations of the latter.

These considerations will enable us to appreciate the definition which Bischoff has adopted, as best expressing the true nature of these deviations from the normal process of growth. "A malformation is then that deviation of form, affecting either an organism or an organ, which is so intimately mixed up with the primitive mode of origin and of development,

* Wagner's Handwörterbuch der Physiologie. Band I. p. 860.

that it can only happen in the earliest period of the embryonic life, or at least before the term of its completion." The author has given a fuller explanation, but in the same sense, in the following words:

"The peculiarity of these malformations, and their essential difference from ordinary morbid changes, are explained by the following considerations:—immediately after birth almost all the organs exist in a condition which, with slight modifications of form, they retain throughout life. All organs, indeed, grow until they are perfectly developed; but this growth is, for the most part, merely a simple augmentation of bulk. A few organs only, as the sexual apparatus and the thymus gland, undergo at a later period comparatively important modifications, either developing themselves more highly, or, on the other hand, disappearing. Indeed, in adults, the changes of the body are, in the normal state, almost solely confined to renewal of material (metamorphosis of tissues), whilst the form of the organs, with very trivial modifications, remains unaltered. The case is different with the embryo and fœtus. Here, as the laws of development teach us, the various parts and organs of the body are gradually developed from the simple stroma of the ovum. During fœtal life we have, therefore, not merely *nutrition*, as afterwards, but also *development;* and whilst, after birth, pathological influences only affect existing structures, or, at most, give rise to the introduction of heterogeneous matters, previous to birth, morbid influences extend their operation even to the development, so that pathological structures are generated, which differ considerably from those occurring after birth." P. 481.

The preceding remarks apply, it must be remembered, only to derangements of form. There are certain other congenital defects which are dependent upon abnormal nutrition, and which, consequently, do not essentially differ from similar disturbances after birth; such as encysted tumours and other morbid products. The more limited instances of abnormal position and excess of parts, belong to distinct classes.

The following classification, adopted by Dr. Vogel, is convenient for studying this department of pathological anatomy, if such it can be called.

"*1st Class.* Malformations, in which certain parts of the normal body are entirely absent, or are too small.—Monstra deficientia.

"*2nd Class.* Malformations produced by fusion or coalescence of organs. Coalitio partium—Symphysis.

"*3rd Class.* Malformations, in which parts in the normal state united—as for instance, in the mesial line of the body—are separated from each other—Clefts, fissures.

"*4th Class.* Malformations, in which normal openings are occluded—Atresia.

"*5th Class.* Malformations of excess, or in which certain parts have attained a disproportionate size—Monstra abundantia.

"*6th Class.* Malformations, in which one or many parts have an abnormal position—Situs mutatus.

"*7th Class.* Malformations of the sexual organs—Hermaphroditism." P. 487.

To these, the author also appends "diseases of the fœtus, and abnormal states of its envelopes."

In a subject so comprehensive and at the same time so full of details as the one under consideration, it is impossible to do more than to offer a few observations relating to some of the most important and interesting points. One of the latter is connected with the causes and mode of origin of twin and triplet monsters, whose bodies are more or less extensively adherent or fused, as it were, together. According to some authorities they arise by a coalescence of two separate germs, which to us appears the more probable explanation; or, they depend, according to others, on a furcation of a single germ. The author, without attempting to decide positively on either of these views, inclines to the latter; and adduces the following as the chief arguments in its favour.

"1. The organs that are united are always similar organs: head with head, thorax with thorax, &c.; a fact that can only be explained in a very forced manner by the assumption of a coalescence of two germs.

"2. There is a complete transition from the cases where two almost perfect individuals are attached at only a circumscribed spot of the body, to those where one individual bears only some trivial supernumerary parts, or other malformation, as, for example, fissure of the skull; in short, to cases whose origin no one would ascribe to a coalescence of two germs.

"3. Finally, it is totally incomprehensible, how, in the case of two separated germs or ova, of which each must have its own membranes, a union of two embryos can take place; and it is just as little to be comprehended how, in such a union, often more than the halves of the two systems can be so intimately fused together, as we sometimes find to be the case. These are the principal reasons which lead me to agree in the opinion, that all twin and triplet monsters, with the exception of the cases of *fœtus in fœtu,* proceed from a simple germ, or ovum." P. 509.

The doctrine we have advocated, to the effect that the larger number of malformations are the result of certain disturbances in the formative process, usually termed "arrest of development," receives the most striking elucidation from congenital defects connected with the termination of the alimentary canal and genital organs. The clue to this part of the inquiry is to be found in three leading facts:—

1. That the posterior extremity of the intestinal passage presents in the early stage of its development a cul-de-sac.

2. That there is subsequently, but still at an early epoch, in both sexes a cloaca, or common connexion of the genito-urinary organs and the intestinal canal.

3. That there are in the two sexes corresponding organs—such as the testes and the ovaria—the penis and the clitoris—the scrotum and the labia majora; and that, in the early development of these parts, they are remarkably similar to each other, so that, till the commencement of the sixth week after conception, the sex cannot be distinguished. It has even been lately affirmed that there is in the male a representative of the uterus, consisting of a median sinus in the prostatic portion of the urethra.

A careful examination of specimens contained in anatomical museums, enlightened by a thorough acquaintance with embryology, will enable the observer to comprehend the true nature of these malformations. Thus, the imperforate anus or the deficiency of the lower extremity of the rectum is evidently dependent on the persistence of the cul-de-sac of the primordial alimentary canal. The specimens in which the rectum communicates, in the male with the urethra and in the female with the vagina, or in either sex with the urinary bladder, is owing to the original cloacal formation remaining, instead of disappearing, as in the normal process, at about the 10th or 11th week, when the canalis uro-genitalis becomes separated from the rectum and anus.

The cases in which the greatest difficulties have arisen, and relating to which the most false notions have prevailed even among medical practitioners, are those that are said to be examples of hermaphroditism.

In former times no doubts seem to have been entertained as to the possibility of the essential organs of generation, by which are meant the testis and the ovarium, co-existing in the same individual. That this was the opinion of Hunter is made evident by his account of the Free Martin, or defective cow-calf. In his introductory observations he says, " as the distinction of male and female parts is natural to most animals; as the union of them in the same animal is also natural to many; and as the separation of them is only a circumstance making no essential difference in the structure of the parts themselves, it becomes no great effort or uncommon play in Nature, sometimes to unite them in those animals in which they are naturally separated; a circumstance we really find takes place in many animals of those orders in which such an union is unnatural." He subsequently expresses his belief that this unnatural hermaphroditism " now and then occurs in every tribe of animals having distinct sexes." Similar views have been prevalent up to a much later period; but it is apparent that, until each successive step in the evolution of the sexual organs in the embryo had been determined, it was not possible justly to interpret the character of the malformations under consideration. The researches of Baer, J. Müller, Rathke, Valentin, and other observers of the present day, which have thrown so much light upon the history of development, have at the same time removed the principal difficulties involved in hermaphroditical conformations, and have necessitated a review *de novo* of the whole question. One of the highest authorities in this branch of anatomical science, Professor Bischoff, and to whose masterly sketch we have already alluded, speaking of the cases formerly recorded, in which male organs were affirmed to exist on one side of the body, and female organs on the other; or in which male and female organs were supposed to be present on both sides in the same individual, judiciously observes, " there are so many probable explanations derived from the history of development as to how these appearances may have originated, partly owing to an arrest of formation, and, partly owing to modifications of individual types of development, that I cannot arrive at an unqualified decision." He afterwards expresses himself more definitely:—" if my view of the so-called hermaphroditical formations be correct, then, strictly speaking, there are none such in the higher animal forms and man; that is, there is no contemporaneous presence of testes and ovaria in one and the same individual: there are, respect being had to these essential organs, only male and female individuals."

We have already said that the complex and peculiar formations connected with the inferior part of the intestinal canal (representing the future rectum), and in an especial manner the complex productions and metamorphoses of the allantois within the body, and of the uro-genital canal, bear intimately upon the question under consideration. It is essential to know, for example, that in the early state of the embryo, the clitoris and the penis have a perfect resemblance to each other; that each possesses a particular groove; that there are in the male cutaneous folds corresponding to the labia majora of the female; that these folds are at first separated by a fissure; and that subsequently they unite to form the scrotum, the prominent suture or raphé indicating their line of junction. The existence, in both sexes, of the peculiar glands called Corpora Wolffiana and the destination of their excretory ducts, are also points of importance; nor should the prostate and Cowper's glands, developed apparently in the female as well as the male, be overlooked. Taking these facts as a guide, it becomes apparent that by far the larger number of the so-called cases of hermaphroditical malformations can at once be referred to certain modifications either of the male or female organs; and doubtless all of them would be thus interpreted, if they were thoroughly investigated. The author is evidently inclined to this opinion, speaking in most doubtful terms of the existence of true hermaphroditism. The most ordinary forms of false hermaphroditism are, in the female, the disproportionate size of the clitoris, so that it may be mistaken for the penis; especially if, which sometimes happens, there is an opening or an inferior channel connected with the clitoris, somewhat resembling the male urethra.

"If, at the same time, as is frequently the case, there is constriction of the vagina, considerable development of the hymen, tumefaction of the labia pudendi, approximation of the total *habitus* to the male sex by deep voice, traces of beard, and slightly developed mammæ; such individuals may be easily mistaken for men." P. 521.

The most common form of "false hermaphroditism in the male sex, arises from the urethra being fissured beneath and atrophied (*hypospadias*), while, at the same time, also, the scrotum and even the perineum are cleft, so that the fissure resembles the female vulva; the resemblance being increased by the circumstance, that, like the latter, it is lined with a soft, red, mucous membrane.

"Generally, also, in such cases, the testicles have not descended (*cryptorchismus*), which adds to the deception, so that such individuals have been frequently taken for girls until the period of puberty, when they have suddenly changed into men." P. 522.

There are some other malformations in the male which have led to a similar error; as where fissure and eversion of the urinary bladder have been mistaken for a vagina; especially if, at the same time, the penis is atrophied and cleft upon its upper side—(*epispadius.*) In rarer cases, the approximation of the male genitals to the female *habitus*, is produced by the penis being attached to the scrotum by adhesions; it is thus drawn downwards, and appears to have vanished; the deception is further favoured when the testicles do not descend.

In dismissing this subject, it will not be superfluous to call attention to the fact, that a large number of these so-called cases of hermaphroditism have been examined only during life, and therefore most inefficiently; that the accounts of them have for the most part been drawn up by persons unacquainted with developmental anatomy; and that, even when the parts have been inspected post-mortem, instead of having been submitted to a rigid scrutiny, the most superficial investigation has satisfied the inquirer, so that such an obvious proceeding, for example, as the application of the microscope to determine whether a doubtful organ was a testis or an ovarium, has not been adopted.

We must here conclude our remarks of Dr. Vogel's Treatise, and although, as already stated, we think there is less of originality than might justly have been expected from a writer who stands so high as a pathologist; and fewer of those philosophic generalizations, which the character of the work would seem to demand, we can strongly recommend the volume now translated to the favourable notice of our readers. In the course of this article, we have not alluded to the English editor, Dr. Day, because, as that gentleman states, the additions he has made are unimportant. We cannot, however, conclude without bearing our testimony to the very efficient manner in which Dr. Day has executed his task; the whole volume indeed, owing to the omission of all German expressions, reads like an original work. The numerous plates, selected principally from the author's "Icones Histologiæ Pathologicæ," form a very important addition to the text, illustrations being an almost indispensable accompaniment of descriptions relating to minute anatomy.

